# Supplementary material for: Effect of Rice Husk-Based Silica on the Friction Properties of High Density Polyethylene Composites
Source: Materials (Basel). 2022 Apr 28;15(9):3191. doi: 10.3390/ma15093191 (PMC9099483; doi:10.3390/ma15093191)
Supplement: Supplementary file 1 [file materials-15-03191-s001.zip › materials-1648915-supplementary.pdf]

Supporting Information

Effect of rice husk-based silica on the friction properties of High Density Polyethylene composites

Yafei Shi [1], Miaomiao Qian [1], Xinru Wang [1], Wanjia Zhang [1], Xuewei Zhang [1], Xiaofeng Wang [2], Yanchao Zhu [1*]

[1] *College of Chemistry, Jilin University, Changchun, 130012, China*

[2] *State Key Laboratory of Inorganic Synthesis & Preparative Chemistry, College of Chemistry, Jilin University, Changchun, 130012, China*

[*] Correspondence: yanchao_zhu@jlu.edu.cn

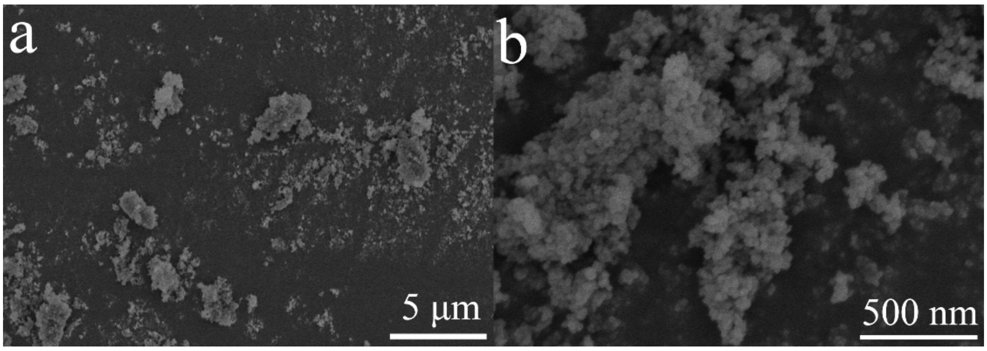

Figure S1. SEM images of commercial silica (SiO_2) at different magnification (**a**) at 5K magnifications and (**b**) at 60K magnifications.

Table S1. Mechanical properties of pure HDPE and composites with SiO_2 as filler.

Samples	Tensile strength (MPa)	Flexural strength (MPa)
HDPE	23.56	18.47
1.5SiO_2/PE	20.74	15.60
3SiO_2/PE	19.26	13.77

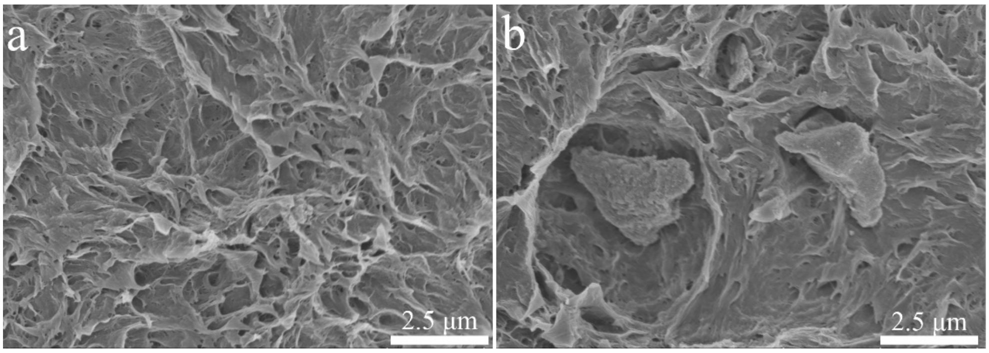

Figure S2. SEM images of HDPE composites brittle fracture surface (**a**) 1.5WRHA/PE and (**b**)

5WRHA/PE.

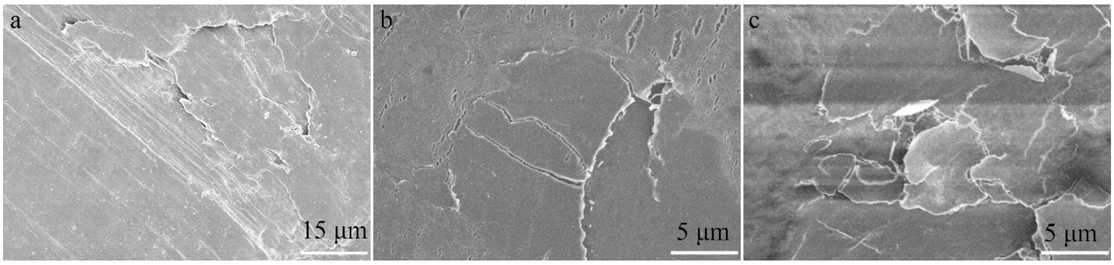

Figure S3. SEM images of worn surface of HDPE composites (**a**) 1.5WRHA/PE, (**b**) 5WRHA/PE and (**c**) 10WRHA/PE.

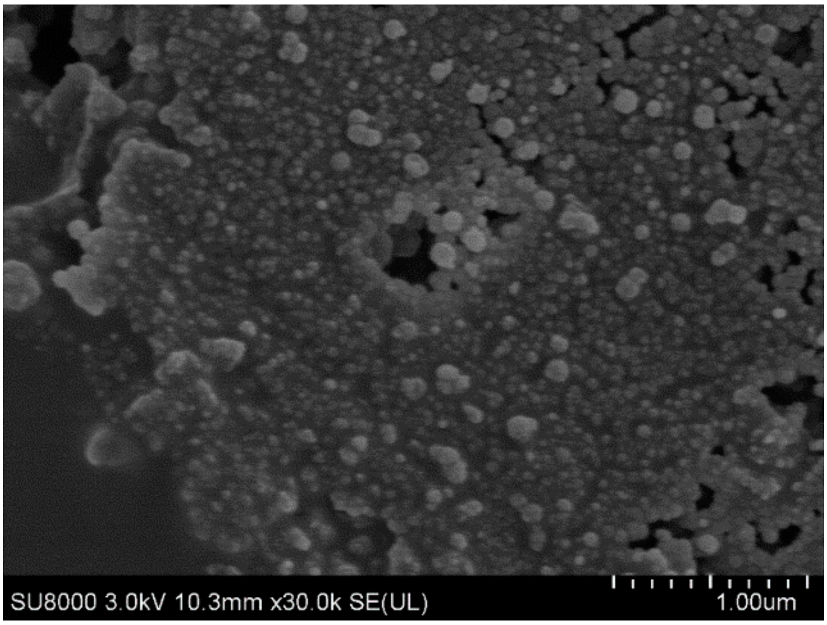

Figure S4. SEM image of ARHA under heat treatment at 800 °C.
